# Ground-Glass Enhancement on Contrast-Enhanced Mammography: A CT-Inspired Qualitative Descriptor for Breast Lesion Characterization

**DOI:** 10.3390/jcm15030999

**Published:** 2026-01-26

**Authors:** Luca Nicosia, Luciano Mariano, Carmen Mallardi, Filippo Pesapane, Mauro Borella, Samuele Frassoni, Vincenzo Bagnardi, Chiara Barizza, Cristian Gialain, Chiara Trentin, Anna Carla Bozzini, Daniele Maiettini, Sonia Santicchia, Enrico Cassano

**Affiliations:** 1 Division of Breast Radiology, Department of Medical Imaging and Radiation Sciences, European Institute of Oncology IRCCS, Via Ripamonti 435, 20141 Milan, Italy; luciano.mariano@ieo.it (L.M.); carmen.mallardi@ieo.it (C.M.); filippo.pesapane@ieo.it (F.P.); anna.bozzini@ieo.it (A.C.B.); enrico.cassano@ieo.it (E.C.); 2 Postgraduation School in Radiodiagnostics, Università degli Studi di Milano, Via Festa del Perdono 7, 20122 Milan, Italy; mauro.borella@unimi.it; 3 Department of Statistics and Quantitative Methods, University of Milano-Bicocca, 20126 Milan, Italy; samuele.frassoni@unimib.it (S.F.); vincenzo.bagnardi@unimib.it (V.B.); 4 Department of Medicine and Surgery, University of Milano-Bicocca, 20126 Milan, Italy; 5 Clinical Trial Office, European Institute of Oncology IRCCS, Via Ripamonti 435, 20141 Milan, Italy; chiara.barizza@ieo.it (C.B.); cristian.gialain@ieo.it (C.G.); 6 Division of Interventional Radiology, European Institute of Oncology IRCCS, Via Ripamonti 435, 20141 Milan, Italy; daniele.maiettini@ieo.it; 7 Radiology Department, Foundation IRCCS Ca’ Granda, Ospedale Maggiore Policlinico, Università Degli Studi di Milano, Via Festa del Perdono 7, 20122 Milan, Italy; sonia.santicchia@policlinico.mi.it

**Keywords:** contrast-enhanced mammography, ground glass enhancement, breast neoplasms, enhancement patterns, lesion conspicuity

## Abstract

**Background:** This study introduces a new qualitative enhancement descriptor for contrast-enhanced mammography (CEM), termed Ground-Glass Enhancement (GGE). The objective was to categorize breast lesions using this descriptor and evaluate its association with malignancy and markers of tumor aggressiveness. **Methods:** In this single-center retrospective study, 249 patients with a single enhancing lesion on CEM were included. Lesions were classified into pure Ground-Glass Enhancement (PGGE), Heterogeneous Ground-Glass Enhancement (HGGE), or Opaque Enhancement (OE) based on the degree of obscuration of the underlying parenchyma. Clinical, imaging, and pathological features were compared across groups. Multivariable logistic regression was used to identify independent predictors of malignancy. **Results:** Significant differences across enhancement patterns were found in lesion conspicuity, enhancement type, size, background enhancement, and patient age. OE lesions more frequently showed high conspicuity (83% vs. 62% in HGGE and 20% in PGGE) and a mass-like appearance (94% vs. 73% in HGGE and 81% in PGGE). HGGE lesions had the largest median size (25 mm, vs. 17 mm in OE and 13 mm in PGGE), and OE lesions most often exhibited minimal background enhancement (77%, vs. 50% in HGGE). In multivariable analysis, mass-like enhancement (OR = 4.59), larger size (OR = 1.27 per +5 mm), and high conspicuity (OR = 3.43) were independently associated with malignancy. Although GGE categories correlated with malignancy in univariable analysis, this was not confirmed in the adjusted model. OE lesions were significantly associated with higher Ki-67 expression (73% with Ki-67 >20%), indicating increased proliferative activity compared with PGGE (43%) and HGGE (57%). **Conclusions:** The GGE descriptor captures clinically relevant imaging features and may support visual stratification of breast lesions on CEM. While not an independent predictor of malignancy, it appears more closely related to markers of tumor aggressiveness.

## 1. Introduction

Breast cancer (BC) is one of the most common malignancies in women and remains a major focus of clinical and translational research. Advances in imaging have substantially improved early diagnosis and patient management [1,2]. Conventional breast imaging—Digital Mammography (DM), Digital Breast Tomosynthesis (DBT), and Ultrasound (US)—continues to represent the cornerstone of routine clinical practice [3,4]. Among these, DM is the only modality proven to reduce BC mortality in population screening, although its sensitivity decreases markedly in women with dense breasts [5,6]. Up to 30% of cancers may be missed in this setting, prompting the integration of DBT and US, particularly in high-risk women [4,7,8]. The need to detect small, biologically aggressive tumors and to better characterize lesion behavior has stimulated growing interest in functional imaging techniques—primarily Magnetic Resonance Imaging (MRI) and Contrast-Enhanced Mammography (CEM)—which have become increasingly available with continuous technological advances [9,10]. CEM combines anatomical and functional information by exploiting the uptake of iodinated contrast agent in relation to tumor angiogenesis, the abnormal neovascularization supporting malignant proliferation [11,12,13]. Technically, it adapts principles of MRI to a radiographic modality by acquiring paired low- and high-energy mammograms following contrast injection [14,15]. Compared with MRI, CEM is more accessible, cost-effective, and generally better tolerated while offering superior diagnostic performance to DM, especially in dense breasts [6,15]. Its enhanced ability to evaluate lesions with suspicious calcifications further increases its diagnostic utility [16]. Historically, a major limitation of CEM had been the absence of a standardized and widely adopted interpretative framework due to its relatively recent incorporation into clinical workflows [17]. This limitation has now been addressed with the release of the updated BI-RADS manual including CEM (ACR, 2025), which formally integrates contrast-enhanced mammography as a dedicated imaging modality with specific descriptors and standardized reporting guidelines [18]. The formal inclusion of CEM within the BI-RADS structure emphasizes its expanding clinical role and highlights the need for consistent, reproducible criteria for image interpretation. However, despite increasing standardization, interpretation of CEM enhancement patterns may still be subject to inter-reader variability, particularly for lesions with subtle or heterogeneous enhancement, underscoring the potential value of visually intuitive qualitative descriptors. Within this evolving diagnostic landscape, we propose a new qualitative descriptor for CEM, inspired by terminology employed in chest Computed Tomography (CT). According to the Fleischner Society, the term “ground-glass opacity” refers to a hazy area of increased lung density through which vascular and bronchial structures remain visible [19]. We adapted this concept to breast imaging by evaluating the degree to which contrast enhancement obscures the underlying breast parenchyma on recombined CEM images [20]. Based on this principle, lesions were categorized as Pure (PGGE), Heterogeneous (HGGE), or Opaque (OGGE) Ground-Glass Enhancement (GGE). To our knowledge, no previous studies have examined the relationship between these enhancement patterns, lesion malignancy, and indicators of tumor aggressiveness. The primary objective of this study was to assess whether specific GGE patterns are associated with malignancy. In confirmed malignant cases, we further evaluated whether these patterns correlate with pathological markers of tumor aggressiveness [21].

## 2. Materials and Methods

This retrospective single-center study was conducted in accordance with the Declaration of Helsinki and approved by the local Ethics Committee (UID 4625).

### 2.1. Study Population

We included all consecutive patients who underwent contrast-enhanced mammography (CEM) for the evaluation of a single enhancing breast lesion and subsequently received a histopathological diagnosis through core needle biopsy or vacuum-assisted biopsy. Lesions were classified as benign or malignant. For malignant lesions, additional pathological information was collected, including tumor grading and receptor status (estrogen receptor, progesterone receptor, HER2, and Ki-67 expression).

### 2.2. Clinical and Imaging Data Collection

Clinical and imaging variables were recorded, including patient age, breast density (ACR categories), type of biopsy, background parenchymal enhancement, lesion morphology, and enhancement pattern (mass vs. non-mass). All CEM examinations were retrospectively reviewed in consensus by three breast radiologists with 10, 5, and 3 years of experience (L.N., L.M., C.M.). Lesions were analyzed according to the CEM descriptors included in the BI-RADS manual (ACR, 2025) [18]. In addition, a dedicated classification system adapted from the chest CT literature on subsolid pulmonary nodules was applied [19]. Since GGE was conceived as an enhancement-based rather than a morphology-based descriptor, both mass and non-mass enhancing lesions were included in the analysis. Three enhancement patterns were defined on recombined CEM images (Table 1): Pure ground-glass enhancement (PGGE)—homogeneous, weak contrast enhancement without focal areas of increased intensity, with clear visualization of the underlying parenchyma; Opaque enhancement (OGGE)—intense contrast enhancement obscuring the underlying parenchymal architecture; and Heterogeneous ground-glass enhancement (HGGE)—mixed uptake with focal areas of higher intensity masking the parenchyma and areas of weak enhancement where parenchymal structures remain visible. These enhancement categories were correlated with histopathological outcomes to assess their association with lesion malignancy. In malignant cases, additional analyses explored the relationship between enhancement pattern and markers of biological aggressiveness.

**Figure 1:**
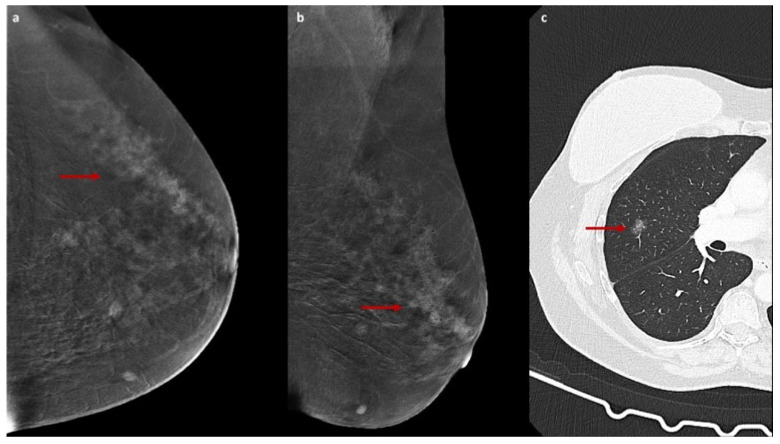
PGGE on CEM with chest CT comparison. Recombined CEM images (**a**,**b**) obtained in a 56-year-old woman for evaluation of suspicious microcalcifications detected on conventional DM show a lesion characterized by pure ground-glass enhancement (PGGE, red arrows), defined as faint and homogeneous contrast agent uptake with preserved visualization of the underlying breast parenchyma. Subsequent histopathological examination after stereotactic biopsy revealed intermediate-grade ductal in situ carcinoma. Chest CT image (**c**) from a 63-year-old patient shows a pure ground-glass nodule (red arrow), classically defined as an area of increased attenuation that does not obscure the underlying bronchial or vascular structures.

**Figure 2:**
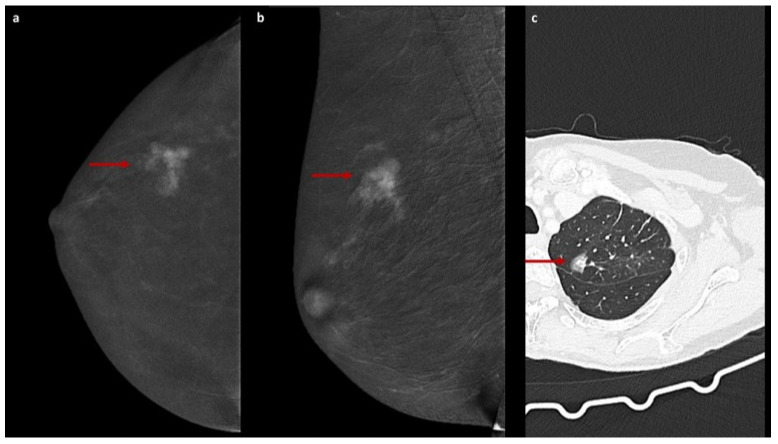
HGGE on CEM with chest CT comparison. Recombined CEM images (**a**,**b**) obtained in a 67-year-old woman for the evaluation of dense breasts in a high-risk clinical setting show a heterogeneous pattern enhancement characterized by mixed contrast agent uptake, with focal areas of higher density partially obscuring the underlying breast parenchyma interspersed with areas of weaker enhancement where parenchymal structures remain visible, consistent with heterogeneous ground-glass enhancement (HGGE, red arrows). Histological examination of an image-guided biopsy demonstrated invasive lobular carcinoma. Chest CT image (**c**) from a 70-year-old patient shows a part-solid ground-glass nodule (red arrow), classically defined as an area of increased attenuation with focal regions of higher density that partially obscure the underlying bronchial or vascular structures.

**Figure 3:**
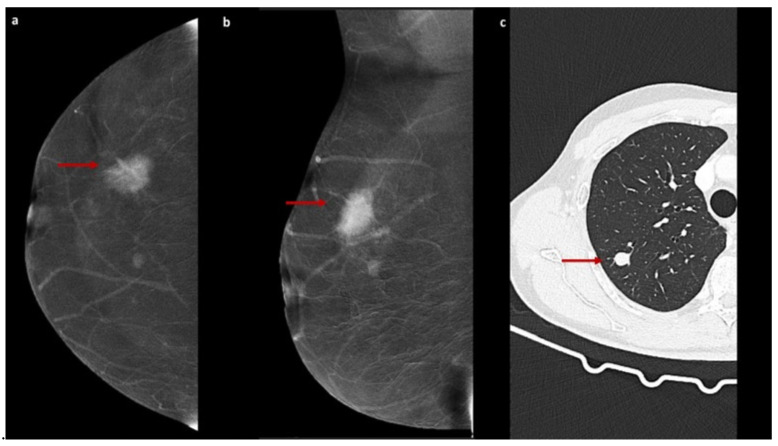
OGGE on CEM with chest CT comparison. Recombined CEM images (**a**,**b**) obtained in a 58-year-old woman referred for further evaluation of a suspicious mass detected on conventional DM show a focal area of intense contrast enhancement with complete obscuring of the underlying breast parenchymal architecture, consistent with opaque enhancement (OGGE, red arrows). The lesion shows high conspicuity and a mass-like pattern enhancement. Histological examination of an image-guided biopsy confirmed the presence of invasive ductal carcinoma. Chest CT image (**c**) from a 65-year-old patient shows a solid pulmonary nodule (red arrow), classically defined as an opacity that completely obscures the underlying bronchial and vascular structures.

The enhancement patterns described above are illustrated in Figure 1, Figure 2 and Figure 3.

### 2.3. Imaging Technique

Dual-energy CEM was performed using one of the following full-field digital systems: Senographe^®^ Essential (GE Healthcare, Chalfont Saint Giles, UK), Amulet^®^ Innovality^®^ (Fujifilm, Tokyo, Japan), or Selenia^®^ Dimensions^®^ (Hologic, Marlborough, MA, USA). An iodinated contrast agent (Visipaque^®^ 320, GE Healthcare, Chalfont St. Giles, UK) was administered intravenously at 1.5 mL/kg using a power injector through an antecubital vein. Two minutes after injection, craniocaudal (CC) and mediolateral oblique (MLO) views were acquired with low-energy (26–31 kVp) and high-energy (45–49 kVp) exposures. Recombined images were obtained through logarithmic subtraction to suppress background parenchyma and enhance the visualization of contrast uptake.

### 2.4. Statistical Analysis

Continuous variables were summarized as medians and ranges; categorical variables as frequencies and percentages. Demographic, clinical, and pathological characteristics were reported overall and stratified by GGE category (PGGE, HGGE, OGGE). Differences among groups were assessed using the Kruskal–Wallis test for continuous variables and Fisher’s exact test for categorical variables. The association between imaging and clinical variables and lesion malignancy was evaluated using univariable logistic regression models. Variables with *p* < 0.05 in univariable analysis were included in a multivariable logistic regression model and quantified by the odds ratios (ORs) with corresponding 95% confidence intervals (CIs). All *p*-values were two-sided, with *p* < 0.05 considered statistically significant. All analyses were performed using SAS software, version 9.4 (SAS Institute, Cary, NC, USA).

## 3. Results

A total of 347 patients with a single suspicious breast lesion who were proposed for pre-biopsy CEM were initially considered for inclusion. Of these, 98 were excluded due to the absence of measurable enhancement on CEM, which precluded GGE classification. The final cohort comprised 249 patients with evaluable contrast media uptake. Lesions were classified into three enhancement patterns based on the GGE descriptor: PGGE in 128 patients (51.4%), HGGE in 86 (34.5%), and OE in 35 (14.1%). The median age of patients at the time of biopsy was 49 years (range, 27–83 years). Most patients had glandular breast tissue (ACR category C, 65.9%), and background parenchymal enhancement was minimal in the majority of cases (63.9%). Lesions were most frequently visualized as masses (80.7%). Patient and lesion characteristics, overall and stratified according to enhancement pattern, are summarized in Table 2.

Several clinical and radiological features showed statistically significant differences across the three groups. Patients with OGGE lesions were significantly older than those in the PGGE and HGGE groups (median ages of 53, 51 and 48 years, respectively; *p* = 0.010). Minimal background enhancement was more frequently observed in OGGE (77%) and PGGE (70%) than in the HGGE group (50%; *p* = 0.008). Enhancement type also varied significantly among the three groups (*p* = 0.022), with mass-type enhancement being most common in OGGE lesions (94%) compared with PGGE (81%) and HGGE lesions (73%). Lesion size differed significantly (*p* < 0.001), with larger lesions observed in the HGGE group (median 25 mm) compared to OGGE lesions (median 17 mm) and PGGE lesions (13 mm). Lesion conspicuity also varied (*p* < 0.001): PGGE lesions were more frequently poorly conspicuous (29% low and 52% moderate), whereas OGGE lesions were predominantly highly conspicuous (83%). Following histopathologic evaluation, 214 of the 249 lesions (85.9%) were confirmed malignant, including both ductal carcinoma in situ (n = 22) and invasive carcinomas (n = 192). The malignancy rate was higher in OGGE (94%) and HGGE (92%) lesions compared with PGGE (80%). In univariable analysis (Table 3), mass-like enhancement was significantly associated with lesion malignancy (vs. non-mass-like: OR = 2.50, 95% CI: 1.14–5.46; *p* = 0.022). Larger lesion size was associated with a higher risk of malignancy (+5 mm: OR = 1.20, 95% CI: 1.03–1.41; *p* = 0.023). The malignancy rate was lower among patients with low or moderate conspicuity (79%) compared with those with high conspicuity (95%; OR = 5.46, 95% CI: 2.04–14.6; *p* < 0.001). Considering PGGE as the reference category, the OR for HGGE was 2.88 (95% CI: 1.19–6.97; *p* = 0.019), and for OGGE it was 4.20 (95% CI: 0.95–18.7; *p* = 0.059).

In the multivariable analysis, mass enhancement (OR = 4.59, 95% CI: 1.73–12.2; *p* = 0.002), lesion size (OR = 1.27 per +5 mm, 95% CI: 1.05–1.54; *p* = 0.012), and high conspicuity (OR = 3.43, 95% CI: 1.14–10.3; *p* = 0.028) remained independent predictors of malignancy. The association between GGE and the outcome was not statistically significant in the multivariable model (OR for HGGE vs. PGGE: 1.52, 95% CI: 0.56–4.13; *p* = 0.41; and for OGGE: 1.35, 95% CI: 0.26–7.03; *p* = 0.72).

Among the biological markers, only Ki-67 expression was significantly associated with GGE type (*p* = 0.008). OGGE lesions more frequently than HGGE and PGGE lesions showed high Ki-67 expression (Ki-67 > 20% in 24/33 OGGE (73%), 45/79 HGGE (57%), and 44/102 PGGE (43%)), indicating increased proliferative activity. No significant associations were found between enhancement pattern and estrogen/progesterone receptor expression (not expressed in 7%, 14%, and 12% of PGGE, HGGE, and OGGE lesions, respectively; *p* = 0.26), HER2 status (positive in 17%, 22%, and 30% of PGGE, HGGE, and OGGE lesions, respectively; *p* = 0.23), or tumor grade (G2/G3 in 83%, 88%, and 94% of PGGE, HGGE, and OGGE lesions, respectively; *p* = 0.25) (Table 4).

## 4. Discussion

This study introduces a new qualitative descriptor for CEM—Ground-Glass Enhancement (GGE)—adapted from the terminology traditionally used to characterize pulmonary nodules on chest CT [19]. Based on the degree of obscuration of the underlying parenchyma on recombined images, three enhancement patterns were defined: PGGE, HGGE, and OGGE. Although GGE was not an independent predictor of malignancy in multivariable analysis, it was associated, in this cohort, with imaging and biological features commonly considered proxies of tumor aggressiveness [22,23]. In particular, OGGE lesions were larger, more conspicuous, and more frequently associated with high Ki-67 expression, suggesting that this visually intuitive descriptor may capture clinically meaningful aspects of tumor biology. These results complement recent evidence showing that high conspicuity on CEM correlates with aggressive tumor subtypes, including HER2-positive and highly proliferative cancers [14]. Parallel findings have long been described in breast MRI, where rapidly enhancing and intensely vascular lesions often reflect more aggressive disease [24,25,26]. A major advantage of the GGE classification is its simplicity. It relies solely on visual assessment and does not require post-processing tools, making it readily applicable in routine practice, as also demonstrated by prior CEM studies focusing on qualitative descriptors [27]. By integrating information on lesion conspicuity, size, and proliferative markers, GGE may assist radiologists in anticipating both the malignant potential and the biological aggressiveness of enhancing lesions, supporting more refined pre-biopsy triage and risk-adapted management strategies [14,22,28,29]. This may be particularly relevant in the context of the recently published BI-RADS 6th Edition (ACR, 2024), which now formally incorporates CEM as a dedicated imaging modality with standardized descriptors [18]. Within this updated framework, additional reproducible qualitative tools such as the GGE may help further refine reporting consistency and diagnostic granularity. The conceptual parallel with chest CT is noteworthy [30]. In pulmonary imaging, the degree of internal solid components within a nodule is strongly linked to invasiveness [31,32,33]. Our findings suggest that an analogous principle—based on the progressive obscuration of background structures—may also hold relevance in breast imaging, although the underlying tissue characteristics differ. Adapting a familiar, visually driven framework to CEM preserves the intuitive nature of ground-glass-type descriptors while tailoring them to the morphologic and contrast properties of breast tissue [30,31,32].

This study has several limitations. Its retrospective, single-center design may limit generalizability, and the proposed classification requires external validation. Prospective multicenter studies will be essential to confirm reproducibility and evaluate the clinical utility of GGE across diverse imaging systems and reader experience levels. Despite these limitations, a key strength of this work is the introduction of a simple, visually recognizable descriptor that may add clinically relevant information to CEM assessment. Similar to lesion conspicuity—which has now been incorporated into the BI-RADS lexicon—the GGE framework captures qualitative enhancement features that are easy to identify yet biologically informative [27,34,35]. By reflecting potential tumor aggressiveness in a reproducible manner, this descriptor may support more confident lesion stratification and contribute to more informed diagnostic and pre-treatment decision-making. Its integration into standardized reporting systems could enrich the interpretative capabilities of CEM and facilitate communication among radiologists and multidisciplinary teams. From a clinical perspective, qualitative GGE patterns may also contribute to interdisciplinary discussions by helping prioritize lesions for biopsy or further assessment, particularly in cases with subtle or equivocal enhancement, although this potential role remains hypothesis-generating.

Inter-observer agreement was not assessed, and therefore the reproducibility of the proposed qualitative GGE descriptor should be confirmed in future studies.

## 5. Conclusions

Although not an independent predictor of malignancy, GGE represents a meaningful and intuitive visual stratification tool in CEM reporting. Its association with markers of tumor aggressiveness underscores its potential to enhance diagnostic confidence, support clinical decision-making, and refine risk stratification within the updated BI-RADS framework for CEM. In addition, the GGE framework may serve as a useful reference for future validation studies and support reader training by providing a visually intuitive approach to enhancement pattern interpretation.

## Figures and Tables

**Table 1:** Correlation between CT Ground-Glass Opacity Patterns and CEM Enhancement Categories.

CT Terminology	CT Definition	Adapted CEM Terminology	CEM Definition
Pure ground-glass nodule	Opacity with increased density that does not obscure underlying vascular or bronchial structures	Pure ground-glass enhancement (PGGE)	Homogeneous, faint contrast agent uptake with preserved visualization of the underlying breast parenchyma
Part-solid nodule	Opacity with focal areas of increased attenuation, not as dense as solid tissue	Heterogeneous ground-glass enhancement (HGGE)	A combination of faint and more intense contrast uptake, with partial obscuration of the underlying parenchyma
Solid nodule	Opacity that completely obscures underlying structures	Opaque enhancement (OGGE)	High-intensity contrast uptake causes loss of visualization of the underlying breast parenchyma

**Table 2:** Patients’ demographic and tumor characteristics, overall and stratified by GGE levels (n = 249).

Variable	Level	Overall (n = 249)	Ground Glass			*p*-Value
			PGGE (n = 128)	HGGE (n = 86)	OGGE (n = 35)	
Age at biopsy, median (min-max)		49 (27–83)	51 (27–83)	48 (30–82)	53 (30–73)	0.010
Background, n (%)	Minimal	159 (63.9)	89 (70)	43 (50)	27 (77)	0.008
	Mild	56 (22.5)	25 (20)	23 (27)	8 (23)	
	Moderate	22 (8.8)	10 (8)	12 (14)	0 (0)	
	Marked	12 (4.8)	4 (3)	8 (9)	0 (0)	
Breast density, n (%)	A	2 (0.8)	1 (1)	0 (0)	1 (3)	0.17
	B	55 (22.1)	33 (26)	16 (19)	6 (17)	
	C	164 (65.9)	84 (66)	55 (64)	25 (71)	
	D	28 (11.2)	10 (8)	15 (17)	3 (9)	
Lesion type, n (%)	Microcalcifications	37 (14.9)	22 (17)	15 (17)	0 (0)	0.069
	Mass	201 (80.7)	99 (77)	68 (79)	34 (97)	
	Mass and microcalcifications	7 (2.8)	4 (3)	2 (2)	1 (3)	
	Architectural distortion	4 (1.6)	3 (2)	1 (1)	0 (0)	
Enhancement type, n (%)	Mass	200 (80.3)	104 (81)	63 (73)	33 (94)	0.022
	Non-mass	49 (19.7)	24 (19)	23 (27)	2 (6)	
Enhancement (mm), median (min–max)		16 (4–96)	13 (4–70)	25 (7–95)	17 (6–96)	<0.001
Lesion conspicuity, n (%)	Low	39 (15.7)	37 (29)	2 (2)	0 (0)	<0.001
	Moderate	103 (41.4)	66 (52)	31 (36)	6 (17)	
	High	107 (43.0)	25 (20)	53 (62)	29 (83)	

**Table 3:** Association of GGE, demographic, and tumor characteristics with lesion malignancy (N = 249).

Variable	Level	Malignancy/Tot (%)	Univariable Analysis			Multivariable Analysis		
			OR	95% CI	*p*-Value	OR	95% CI	*p*-Value
Overall	-	214/249 (85.9)	-	-	-	-	-	-
Ground glass	PGGE	102/128 (80)	Ref.	-	-	Ref.	-	-
	HGGE	79/86 (92)	2.88	1.19–6.97	0.019	1.52	0.56–4.13	0.41
	OGGE	33/35 (94)	4.20	0.95–18.7	0.059	1.35	0.26–7.03	0.72
Age at biopsy	+5 years		0.96	0.82–1.13	0.61			
Background	Minimal/Mild	186/215 (87)	Ref.	-	-			
	Moderate/Marked	28/34 (82)	0.73	0.28–1.91	0.52			
Breast density	A/B	50/57 (88)	Ref.	-	-			
	C/D	164/192 (85)	0.82	0.34–1.99	0.66			
Enhancement type	Non-mass	37/49 (76)	Ref.	-	-	Ref.	-	-
	Mass	177/200 (89)	2.50	1.14–5.46	0.022	4.59	1.73–12.2	0.002
Enhancement (mm)	+5 mm		1.20	1.03–1.41	0.023	1.27	1.05–1.54	0.012
Lesion conspicuity	Low/Moderate	112/142 (79)	Ref.	-	-	Ref.	-	-
	High	102/107 (95)	5.46	2.04–14.6	<0.001	3.43	1.14–10.3	0.028

**Table 4:** Association of GGE with biomarkers and tumor grade among patients with confirmed malignant lesions (N = 214).

Variable	Level	Overall (N = 214)	Ground Glass			*p*-Value
			PGGE (n = 102)	HGGE (n = 79)	OGGE (n = 33)	
ER/PgR, N (%)	Not expressed (Both 0)	22 (10.3)	7 (7)	11 (14)	4 (12)	0.26
	Incompletely or highly expressed (ER > 0 or PgR > 0)	192 (89.7)	95 (93)	68 (86)	29 (88)	
Ki-67, N (%)	≤20%	101 (47.2)	58 (57)	34 (43)	9 (27)	0.008
	>20%	113 (52.8)	44 (43)	45 (57)	24 (73)	
HER2, N (%)	Positive	44 (20.6)	17 (17)	17 (22)	10 (30)	0.23
	Negative	170 (79.4)	85 (83)	62 (78)	23 (70)	
Grade, N (%)	G1	28 (13.2)	17 (17)	9 (12)	2 (6)	0.25
	G2/G3	184 (86.8)	84 (83)	69 (88)	31 (94)	
	Missing	2	1	1	0	

## Data Availability

The data presented in this study are available on reaseonable request from the corresponding author.
